# Contribution of a family 1 carbohydrate-binding module in thermostable glycoside hydrolase 10 xylanase from *Talaromyces cellulolyticus* toward synergistic enzymatic hydrolysis of lignocellulose

**DOI:** 10.1186/s13068-015-0259-2

**Published:** 2015-05-13

**Authors:** Hiroyuki Inoue, Seiichiro Kishishita, Akio Kumagai, Misumi Kataoka, Tatsuya Fujii, Kazuhiko Ishikawa

**Affiliations:** Research Institute for Sustainable Chemistry, National Institute of Advanced Industrial Science and Technology (AIST), 3-11-32 Kagamiyama, Higashi-Hiroshima, Hiroshima 739-0046 Japan

**Keywords:** Enzymatic hydrolysis, GH10 xylanase, Cellulase, Family 1 carbohydrate-binding module, Lignocellulosic biomass, Rice straw, *Talaromyces cellulolyticus*

## Abstract

**Background:**

Enzymatic removal of hemicellulose components such as xylan is an important factor for maintaining high glucose conversion from lignocelluloses subjected to low-severity pretreatment. Supplementation of xylanase in the cellulase mixture enhances glucose release from pretreated lignocellulose. Filamentous fungi produce multiple xylanases in their cellulase system, and some of them have modular structures consisting of a catalytic domain and a family 1 carbohydrate-binding module (CBM1). However, the role of CBM1 in xylanase in the synergistic hydrolysis of lignocellulose has not been investigated in depth.

**Results:**

Thermostable endo-β-1,4-xylanase (Xyl10A) from *Talaromyces cellulolyticus*, which is recognized as one of the core enzymes in the fungal cellulase system, has a modular structure consisting of a glycoside hydrolase family 10 catalytic domain and CBM1 at the C-terminus separated by a linker region. Three recombinant Xyl10A variants, that is, intact Xyl10A (Xyl10Awt), CBM1-deleted Xyl10A (Xyl10AdC), and CBM1 and linker region-deleted Xyl10A (Xyl10AdLC), were constructed and overexpressed in *T. cellulolyticus*. Cellulose-binding ability of Xyl10A CBM1 was demonstrated using quartz crystal microbalance with dissipation monitoring. Xyl10AdC and Xyl10AdLC showed relatively high catalytic activities for soluble and insoluble xylan substrates, whereas Xyl10Awt was more effective in xylan hydrolysis of wet disc-mill treated rice straw (WDM-RS). The enzyme mixture of cellulase monocomponents and intact or mutant Xyl10A enhanced the hydrolysis of WDM-RS glucan, with the most efficient synergism found in the interactions with Xyl10Awt. The increased glucan hydrolysis yield exhibited a linear relationship with the xylan hydrolysis yield by each enzyme. This relationship revealed significant hydrolysis of WDM-RS glucan with lower supplementation of Xyl10Awt.

**Conclusions:**

Our results suggest that Xyl10A CBM1 has the following two roles in synergistic hydrolysis of lignocellulose by Xyl10A and cellulases: enhancement of lignocellulosic xylan hydrolysis by binding to cellulose, and the efficient removal of xylan obstacles that interrupt the cellulase activity (because of similar binding target of CBM1). The combination of CBM-containing cellulases and xylanases in a fugal cellulase system could contribute to reduction of the enzyme loading in the hydrolysis of pretreated lignocelluloses.

## Background

Lignocellulose is one of the most abundant organic compounds in the biosphere and contains large amounts of polysaccharides, such as cellulose and hemicellulose, which serve as the source of fermentable sugars used in the production of biofuels or chemicals. An efficient enzymatic hydrolysis of the cellulose and hemicellulose components to fermentable sugars is a key step in the bioconversion of lignocellulose [1,2]. The digestibility of these components is increased by pretreatment processes such as dilute acid, alkali, hot compressed water, and milling treatments that disrupt the rigid structural network consisting of cellulose-hemicellulose-lignin [3]. In the current industrial biofuel production from lignocellulose, the use of low-severity pretreatment without chemical addition is expected to have advantages that result in lowering the capital cost and minimizing waste generation [4]. However, low-severity factor results in less sugar yield. The residual hemicellulose has been known to limit the hydrolysis of cellulose, which has a β-1,4-glucan structure [5-8]. Therefore, an efficient enzymatic removal of hemicellulose is an important factor to maintain high glucose conversion from lignocellulose subjected to the low-severity pretreatment.

Xylan is a major polysaccharide of hemicellulose from agricultural residues and hardwoods, consisting of a linear backbone of β-D-xylopyranosyl residues linked by β-1,4-glycosidic bonds. Most of the xylan found is heteroxylan and its backbone could be branched due to substitution of different side groups such as L-arabinose, D-galactose, acetyl, feruloyl, *p*-coumaroyl, and glucuronic acid residues. Xylan forms an overlying layer through hydrogen bonding with the cellulose, while it is covalently linked with lignin, which forms an outer sheath to protect the plant [9]. Therefore, complete enzymatic hydrolysis of xylan requires a large number of hemicellulases and accessory enzymes with different specificities [9]. Xylanases (endo-β-1,4-xylanase; EC 3.2.1.8) are the main enzymes that cleave the β-1,4-glycosidic bonds in the xylan backbone. Supplementation of xylanase to cellulase mixture has been reported to enhance glucose release from the pretreated lignocellulose [6-8,10,11], which in turn also dramatically reduces cellulase loading needed to achieve reasonable glucan hydrolysis yields from the cellulose [8]. These synergistic interactions observed between the cellulases and xylanases are believed to result from an increase in the accessibility of the cellulases to cellulose due to removal of the hemicellulose [6,8,11]. The synergistic action of xylanases along with certain accessory enzymes that act on the removal of xylan side chains also contribute to further hydrolysis of cellulose by cellulase-xylanase interactions [2]. The optimization of these enzymes for the pretreated lignocellulose has great potential not only for enhancing the glucose and xylose yields but also for reducing enzyme loading and cost [1,12,13].

Based on the amino-acid sequence similarities, most xylanases are classified into glycoside hydrolase family 10 (GH10) that have a (β/α)_8_ TIM-barrel structure and family 11 (GH11) that consists of a β-jelly roll structure [14-16]. GH11 xylanases have been recognized as important enzymes capable of enhancing biomass hydrolysis due to their ability to effectively hydrolyze various xylanolytic substrates [10]. On the other hand, GH10 xylanases exhibit lower substrate specificity in comparison to the GH11 xylanases and possess a higher affinity to the highly branched xylan backbone [14]. A combination of GH10 and 11 xylanases in the cellulase cocktail help in increasing both the glucose and xylose yields in ammonia fiber expansion-pretreated corn stover [12,13]. Furthermore, it has been reported that GH10 xylanases are more effective than GH11 xylanases for the synergistic glucan hydrolysis of the assessed pretreated lignocellulosic substrates [10,17]. These studies suggest that GH10 xylanases could be better candidates for enhancing the hydrolysis of more realistic substrates.

Filamentous fungi are the major source of industrial enzymes and produce a variety of cellulases and hemicellulases with high productivity and catalytic efficiency, both of which are required for low-cost enzyme supply [1]. Multiple xylanases are produced in the fungal cellulase system and a large number of GH10 xylanases found in basidiomycota and ascomycota have a modular structure that include a family 1 carbohydrate-binding module (CBM1) at N- and C-termini [18,19]. CBM1s are exclusively found in fungal cellulases and hemicellulases and generally target the enzymes to the cellulose surface. The major function of CBM is to increase the effective enzyme concentration on the polysaccharide surface and thus, enhance the enzymatic activity [20,21]. It is known that removal of the CBM1 significantly reduces the activity of cellulases toward insoluble substrates [20,22]. On the other hand, the presence of CBM1 in two GH10 xylanases from *Myceliophthora thermophila* was not always beneficial in the degradation of soluble and insoluble xylan substrates [23]. However, the role of CBM1 in xylanase in the synergistic hydrolysis of lignocellulose has not been explored in detail.

In a previous study, we identified a thermostable GH10 xylanase (Xyl10A) in the *Talaromyces cellulolyticus* cellulase system as one of the core enzymes for the hydrolysis of lignocellulose [24]. Xyl10A is a modular enzyme consisting of an N-terminal catalytic domain and CBM1 separated by a Ser/Thr-rich linker region [19]. Furthermore, an increase in the synergistic glucan hydrolysis of pretreated corn stover by the mixture of cellobiohydrolase I (Cel7A), cellobiohydrolase II (Cel6A), and Xyl10A from *T. cellulolyticus* has been observed [24]. The purpose of this study is to evaluate the role of Xyl10A CBM1 in the synergistic hydrolysis of the lignocellulose substrate. The wet disc-mill treated rice straw (WDM-RS) was used as the substrate that was subjected to synergistic hydrolysis using a mixture of cellulase monocomponents and Xyl10A, with or without the CBM1. The efficacies of the cellulose-binding ability of Xyl10A CBM1 for efficient synergistic hydrolysis of lignocellulose are discussed in this study.

## Results

### Expression and purification of recombinant enzymes: Xyl10Awt, Xyl10AdC, and Xyl10AdLC

**Figure 1:**
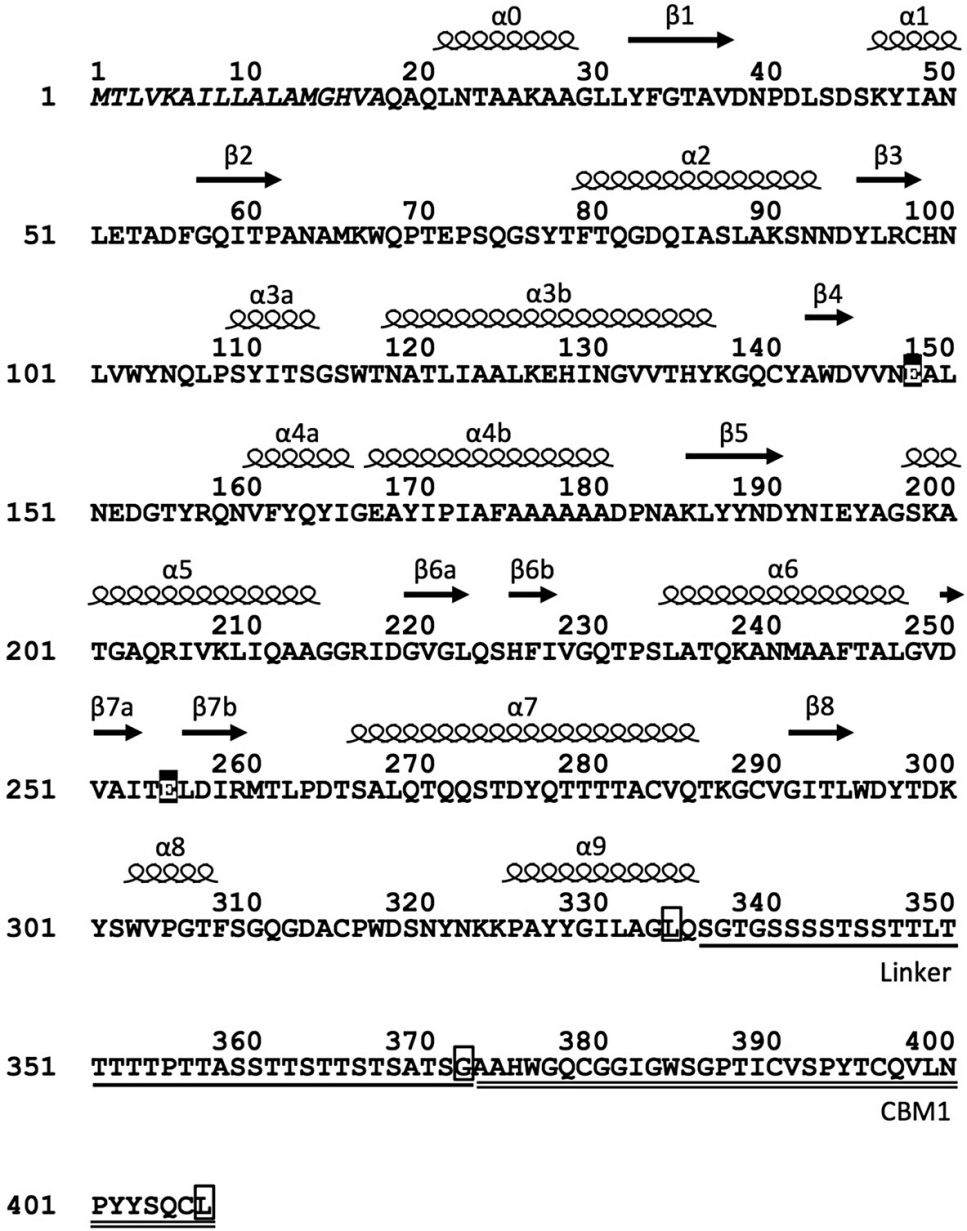
Amino acid sequence of Xyl10A with predicted secondary structural elements. The putative signal sequence is shown in *italics*. The putative domains in mature Xyl10A are as follows: catalytic domain (amino-acid residues 20 to 335); Ser/Thr-rich linker region (336 to 372), *underlined*; CBM1 (373 to 407), *double underlined*. C-terminal residues of Xyl10AdLC (Leu334), Xyl10AdC (Gly372), and Xyl10Awt (Leu407) are enclosed in a *box*. The predicted catalytic residues are shown in filled *black boxes*. The predicted secondary structural elements (α-helices and β-strands) based on the structure of a GH10 xylanase from *Fusarium oxysporum* (FoXyn10a, sequence identity, 50%, PDB ID, 3U7B) as template [50] are indicated above the sequence.

**Figure 2:**
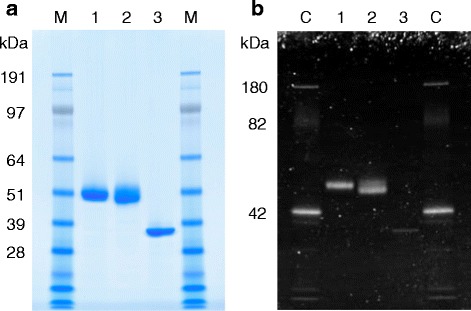
SDS-PAGE (4% to 12%) of purified intact and mutant Xyl10As. Approximately 5 μg **(a)** and 0.7 μg **(b)** of proteins were loaded on each gel and stained with Colloidal Coomassie G-250 and Pro-Q Emerald glycoprotein stain, respectively. Glycoproteins were visualized using a 300-nm UV transilluminator. Lanes: 1, Xyl10Awt; 2, Xyl10AdC; 3, Xyl10AdLC; M*,* SeeBlue Plus2 protein standard (Invitrogen); C*,* CandyCane glycoprotein marker (Invitrogen).

To evaluate the role of CBM1 in Xyl10A from *T. cellulolyticus*, three recombinant forms of Xyl10A were designed, that is, Xyl10Awt, an intact enzyme; Xyl10AdC, a CBM1-deleted form that has Gly372 as the C-terminal residue; and Xyl10AdLC, a CBM1 and linker region-deleted form that has Leu334 as the C-terminal residue (Figure 1). These three recombinant enzymes were secreted extracellularly using the *T. cellulolyticus* homologous expression system under the control of the starch-inducible glucoamylase promoter [19]. The analysis of culture supernatant showed these recombinant proteins as major bands in the SDS-PAGE analysis (data not shown). Our earlier work has shown that the recombinant Xyl10Awt had similar physicochemical and enzymatic properties as the native Xyl10A [19]. The apparent molecular weights for purified recombinant Xyl10As were estimated to be 51 k (Xyl10Awt), 50 k (Xyl10AdC), and 37 k (Xyl10AdLC) on SDS-PAGE gel (Figure 2a). The molecular weights of Xyl10Awt and Xyl10AdC were higher in comparison to the calculated molecular masses, that is, 41,490 and 37,747 Da, respectively, whereas the molecular weight of Xyl10AdLC was nearly identical to the calculated value (34,245 Da). The result of glycosylation staining of these enzymes suggested that the Ser/Thr-rich linker regions of Xyl10Awt and Xyl10AdC are highly modified by *O*-glycosylation (Figure 2b), as reported for fungal cellulolytic enzymes possessing linker and CBM1 [25]. The similar values for molecular weight between Xyl10Awt and Xyl10AdC suggest that Xyl10AdC is slightly more glycosylated in comparison to Xyl10Awt.

### Binding of Xyl10A to cellulose

CBM1 is the most common cellulose-binding module observed in fungal cellulases and various hemicellulases. CBMs in several hemicellulases, such as GH5 β-mannanase, GH74 xyloglucanase, GH54, and GH62 α-L-arabinofuranosidase, have been also experimentally demonstrated to target binding to cellulose [26-29]. The cellulose-binding ability of a GH10 xylanase CBM1 has recently been reported for an enzyme from *Malbranchea pulchella* [30]. Xyl10A CBM1 also contains the following highly conserved residues within its amino-acid sequence (Figure 1): three aromatic residues (Trp376, Tyr402, and Tyr403) that are hypothesized to form the binding face for cellulose, two polar residues (Gln378 and Asn400) that potentially form hydrogen bonds with cellulose, and four cysteine residues involved in disulfide bonds (Cys379, Cys390, Cys396, and Cys406) [29,31].

**Figure 3:**
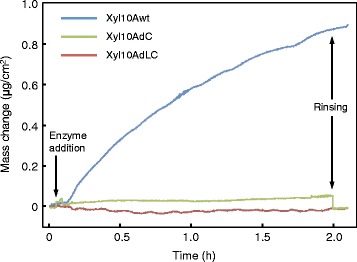
Adsorption of intact and mutant Xyl10As onto the cellulose nanocrystal films. Enzyme solution (250 μM) in 50 mM sodium acetate buffer (pH 5.0) was added into the QCM-D flow cell at a flow rate of 50 μL/min for 10 min. The frequency change due to adsorption on cellulose nanocrystal film was recorded and converted into adsorbed mass density (μg/cm^2^).

We used a quartz crystal microbalance with the dissipation monitoring (QCM-D) technique to compare the binding affinities of intact and mutant Xyl10As toward crystalline cellulose. It has been established that QCM-D is a reliable method to evaluate the binding affinity of an enzyme toward its substrate [32,33]. Figure 3 shows the results of binding assays between a cellulose nanocrystal film and the three purified Xyl10As. As expected, an increase in mass was observed for Xyl10Awt due to adsorption of the enzymes to the cellulose surface, indicating that Xyl10A CBM1 has binding ability to crystalline cellulose. Adsorption of Xyl10Awt on cellulose continued to increase under the presence of 250 μM enzyme concentration during the experimental time of 2 hours. In addition, Xyl10Awt was retained on the surface of crystalline cellulose even after rinsing with buffer solution. However, the small mass change observed for Xyl10AdC decreased after rinsing and thereafter was consistent with the expected mass value of Xyl10AdLC. This observation suggests that the Ser/Thr-rich linker region in Xyl10AdC shows weak interactions with the cellulose surface.

### Enzymatic properties of intact and mutant Xyl10As

The substrate specificities of Xyl10As with and without CBM1 were examined on various substrates, including both soluble and insoluble xylan and chromogenic compounds (Table 1). All Xyl10As showed the highest activities for arabinoxylan and detectable activities for the chromogenic compounds. This observation suggests that the Xyl10A catalytic domain has catalytic versatility and hydrolyzes short xylooligomers (as is seen for other GH10 xylanases) [16,18]. Further, no activity was detected when Avicel, carboxymethyl cellulose, and xyloglucan were used as substrates. Deletion of CBM1 resulted in an increase in the hydrolytic activities of Xyl10As for all the soluble substrates and insoluble oat-spelt xylan. The specific activities of Xyl10AdC and Xyl10AdLC for these substrates were found to be 1.2- to 1.6-fold higher than those of Xyl10Awt at 45°C (Table 1). These results indicate that the presence of CBM1 had no direct effects on xylan degradation.

**Table 1:** **Substrate specificities of purified intact and mutant Xyl10As toward different substrates**

**Substrates**	**Xyl10Awt (A) (U/nmol)**	**Xyl10AdC (B) (U/nmol)**	**Xyl10AdLC (C) (U/nmol)**	**(B)/(A)**	**(C)/(A)**
Birchwood xylan	10.6	15.1	13.9	1.42	1.31
Wheat arabinoxylan	21.9	35.5	28.2	1.62	1.29
Insoluble oat-spelt xylan	2.53	3.85	3.66	1.52	1.45
PNP-lactose	0.048	0.064	0.061	1.34	1.28
PNP-cellobiose	0.251	0.393	0.342	1.56	1.36
PNP-xylose	0.022	0.028	0.026	1.25	1.18
WDM-RS^a^	0.094	0.054	0.066	0.57	0.70

Activities were determined at 45°C (pH 5.0). ^a^Activities for WDM-RS were determined at 50°C (pH5.0). PNP-lactose, *p*-nitrophenyl-β-D-lactoside; PNP-cellobiose, *p*-nitrophenyl-β-D-cellobioside; PNP-xylose, *p*-nitrophenyl-β-D-xyloside; WDM-RS, wet disc-mill treated rice straw.

**Figure 4:**
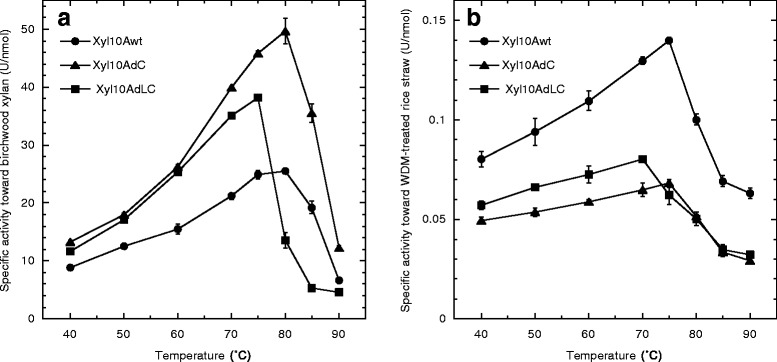
Effect of temperature on xylanase activities of intact and mutant Xyl10As. The enzyme reaction was carried out in 50 mM sodium acetate buffer (pH 5.0) for **(a)** 10 min for 1% (w/v) birchwood xylan with 24 nM to 29 nM enzyme and **(b)** 60 min for 3% (w/v) WDM-RS with a 1.2 μM to 1.5 μM enzyme at different temperatures. The values of specific activities are the mean of three replicates.

The activity of mutant enzymes was significantly enhanced at higher temperature. Although the Xyl10Awt and Xyl10AdC have the same optimum temperature (80°C) for xylanase activity, the specific activities of Xyl10AdC were increased by 1.9-fold for birchwood xylan when the temperature increased from 70°C to 80°C (Figure 4a). The optimum activity for Xyl10AdLC was found at 75°C and dropped dramatically with increase in temperature up to 80°C. The mutant enzymes showed relatively higher activities in comparison to Xyl10Awt under acidic conditions (data not shown). The optimum pH for the hydrolysis of birchwood xylan by mutant Xyl10As was around pH 4.5 in McIlvaine buffer, whereas that for Xyl10Awt was around pH 5.0. The relative activities of Xyl10Awt, Xyl10AdC, and Xyl10AdLC at pH 3.4 were 33%, 78%, and 67%, respectively, for the optimum pH conditions. The kinetic parameters for Xyl10As were determined using birchwood xylan as substrate. The *k*_cat_ for mutant enzymes was 1.3- to 1.5-fold higher in comparison to that of Xyl10Awt, whereas the apparent *K*_m_ values were almost of the same magnitude in all the Xyl10As (Table 2). Therefore, it was concluded that Xyl10As without CBM1 are more efficient (*k*_cat_/*K*_m_) in comparison to Xyl10Awt for easily degradable substrates such as purified model xylan.

**Table 2:** **Kinetic parameters for hydrolysis of birchwood xylan by intact and mutant Xyl10As**

**Enzyme**	***K***_**m**_ **(mg mL** ^**−1**^ **)**	***k***_**cat**_ **(s** ^**−1**^ **)**	***k***_**cat**_ **/** ***K***_**m**_ **(s** ^**−1**^ **mg** ^**−1**^ **mL** ^**−1**^ **)**
Xyl10Awt	2.51 ± 0.42	232 ± 12.4	92.2 ± 16.2
Xyl10AdC	2.80 ± 0.18	359 ± 7.8	128 ± 8.9
Xyl10AdLC	2.59 ± 0.14	310 ± 5.4	120 ± 6.7

Reactions were performed at 45°C (pH 5.0).

In contrast, the Xyl10Awt was more effective in the degradation of residual xylan in insoluble lignocellulose substrate than mutant enzymes. When WDM-RS was used as the lignocellulose substrate, Xyl10Awt showed relatively higher xylanase activity, that is, 1.7- and 1.4-fold in comparison to Xyl10AdC and Xyl10AdLC, respectively (Table 1). Thus, this result suggests that the binding of the enzyme to cellulose through CBM1 indirectly enhances the xylan degradation in WDM-RS. Xyl10Awt had higher activity compared to mutant enzymes at all examined temperatures (Figure 4b). The activity of Xyl10AdC was found to be relatively lower compared to Xyl10AdLC (Figure 4b), suggesting that presence of the linker region without CBM1 may have a negative impact on xylan degradation in WDM-RS.

Xyl10A from *T. cellulolyticus* is a thermostable GH10 xylanase having a thermal midpoint (*T*_m_) above 80°C [19,24]. *T*_m_ values of intact and mutant Xyl10As measured by fluorescence-based thermal shift assay were estimated to be 81.0°C (Xyl10Awt), 81.0°C (Xyl10AdC), and 75.5°C (Xyl10AdLC), and these values were in good agreement with the results of optimum temperature for xylanase activities of these enzymes (Figure 4). The reason behind the relatively low thermostability of Xyl10AdLC may be due to an incomplete α9 helix at the C-terminal region (Figure 1).

### Synergistic hydrolysis of pretreated rice straw

WDM-RS is a fine fiber material generated by mechanical fibrillation of rice straw soaked in water and has been reported to have improved hydrolysis yield by the cellulase mixture [34]. However, the reduction of crystallinity and removal of major hemicellulose fraction were not observed in the pretreated rice straw sample [34]. The potential of intact and mutant Xyl10As, along with cellulase monocomponents consisting of purified Cel7A and Cel6A from *T. cellulolyticus*, was evaluated for the hydrolysis of WDM-RS glucan and xylan. Optimal WDM-RS glucan hydrolysis by the mixture of Cel7A and Cel6A was observed at a mole ratio of approximately 3.9:4.5 (data not shown), and hence, the mixture of Cel7A (38.9 nmol/g glucan) and Cel6A (44.5 nmol/g glucan) were replaced with various ratios of Xyl10Awt (0 to 98.4 nmol/g glucan), Xyl10AdC (0 to 108 nmol/g glucan), and Xyl10AdLC (0 to 119 nmol/g glucan). In addition, a purified GH3 β-glucosidase (Bgl3A) from *T. cellulolyticus* was also added to the reaction mixture to prevent inhibition by cellobiose at 6.17 nmol/g glucan of protein loading.

**Figure 5:**
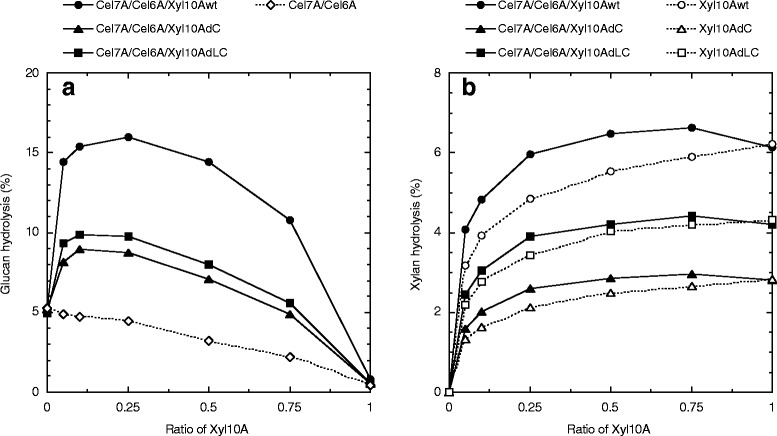
Hydrolysis of WDM-RS glucan and xylan using different ratios of cellulase monocomponents and xylanases. Hydrolysis experiments were carried out at pH 5.0, 45°C with 3% (w/v) WDM-RS. The mixture consisting of Cel7A (38.9 nmol/g glucan) and Cel6A (44.5 nmol/g glucan) was replaced with various ratios of Xyl10Awt (0 to 98.4 nmol/g glucan), Xyl10AdC (0 to 108 nmol/g glucan), and Xyl10AdLC (0 to 119 nmol/g glucan) in a final volume of 1 mL (*filled symbols*). Bgl3A (6.17 nmol/g glucan) was added in all reaction mixtures. The cellulases or Xyl10A in the reaction mixture was substituted by 50 mM sodium acetate buffer (pH 5.0) as a control (*empty symbols*). The hydrolysis yields of **(a)** WDM-RS glucan and **(b)** xylan were calculated from the amount of glucose and the total amount of xylose and xylobiose, respectively, released in the hydrolysate after 24 h.

When intact or mutant Xyl10A was used to replace small amounts of cellulases, a significant increase in the degree of synergism was observed in the hydrolysis of WDM-RS glucan after 24-h reaction (Figure 5a). Further, the mixture containing Xyl10wt showed greater increase in the glucan hydrolysis by cellulases in comparison to the mutant Xyl10As. The highest glucan hydrolysis yields were observed at ratios of 0.25 (Xyl10Awt), 0.1 (Xyl10AdC), and 0.1 (Xyl10AdLC), which corresponded to a 3.1-, 1.7-, and 1.9-fold increase in the glucan hydrolysis yield, respectively, with respect to the corresponding cellulase loading without Xyl10A (Figure 5a). The synergistic enhancement of glucan hydrolysis was observed with an increase in the ratio of Xyl10A. The greatest enhancement of glucan hydrolysis was observed at a ratio of intact or mutant Xyl10A of 0.75, and the fold increases in the glucan hydrolysis yields were 4.9-fold (Xyl10Awt), 2.2-fold (Xyl10AdC), and 2.5-fold (Xyl10AdLC) with respect to the corresponding cellulase loading without Xyl10A (Figure 5a). These enzyme mixtures also showed the highest xylan hydrolysis yields for WDM-RS (Figure 5b). These results indicate that the synergistic interaction between cellulases and Xyl10A is closely correlated with the xylan removal from WDM-RS. Therefore, Xyl10A CBM1 that increases the xylan degradation in WDM-RS seems to be playing an important role in the synergistic hydrolysis of WDM-RS. The enhancement of glucan hydrolysis showed a similar trend after 96 h for WDM-RS, and the highest glucan hydrolysis was obtained at a ratio of intact or mutant Xyl10A of 0.25 (data not shown). In this case, the glucan hydrolysis yields showed an increase to 28% (Xyl10Awt), 18% (Xyl10AdC), and 21% (Xyl10AdLC).

The synergistic interaction between cellulases and a Xyl10A variant slightly enhanced the xylan hydrolysis of WDM-RS. A 1.1- to 1.2-fold increase in xylan hydrolysis yield was observed at a ratio of intact or mutant Xyl10A of 0.25 in comparison to the corresponding Xyl10A loading without cellulases (Figure 5b). The total sugar hydrolysis yield of both glucan and xylan was most effective at a ratio of intact or mutant Xyl10A of 0.25. The total yields after 96 h reaction were 21% (Xyl10Awt), 13% (Xyl10AdC), and 16% (Xyl10AdLC) due to relatively low xylan hydrolysis yields (4.4% to 10.4%). Glucose, xylose, and xylobiose were observed in the hydrolysates. The amounts of xylobiose released in the hydrolysates were 1.5- to 1.65-fold higher than those of xylose.

### Implications of xylan removal for synergistic glucan hydrolysis in WDM-RS

**Figure 6:**
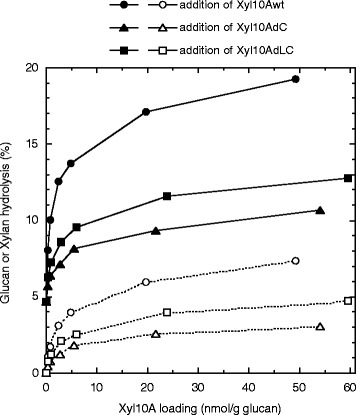
Effect of intact or mutant Xyl10A supplements on WDM-RS hydrolysis by cellulase monocomponents. Intact or mutant Xyl10As were added to the fixed amounts of the mixture of Cel7A (38.9 nmol/g of glucan), Cel6A (44.5 nmol/g of glucan), and Bgl3A (6.17 nmol/g of glucan). Hydrolysis experiments were carried out at pH 5.0, 45°C with 3% (w/v) WDM-RS in a final volume of 1 mL. The hydrolysis yields of WDM-RS glucan (*filled symbols*) and xylan (*empty symbols*) were calculated from the amount of glucose and the total amount of xylose and xylobiose, respectively, released in the hydrolysate after 24 h.

**Figure 7:**
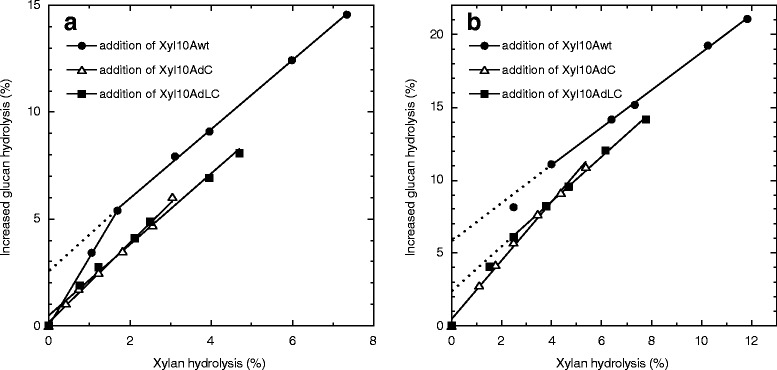
Glucose release and xylan removal in WDM-RS hydrolysis by cellulase monocomponents with differing xylanase supplementation. The relationship between increased glucan hydrolysis and xylan hydrolysis was analyzed using the hydrolysate after 24 h **(a)** and 96 h **(b)** based on hydrolysis experiments described in Figure 6.

To evaluate the outcomes of xylan removal from cellulose on glucan hydrolysis of WDM-RS, the experiments were carried out over a range of supplementations of Xyl10Awt (0 to 49.2 nmol/g of glucan), Xyl10AdC (0 to 54.1 nmol/g of glucan), and Xyl10AdLC (0 to 59.6 nmol/g of glucan) in the presence of fixed amounts of the mixture of Cel7A (38.9 nmol/g of glucan), Cel6A (44.5 nmol/g of glucan), and Bgl3A (6.17 nmol mg/g of glucan) at 45°C for 24 h. Glucan and xylan hydrolysis yields increased with increasing xylanase loadings. These yields were significantly increased at a slight xylanase loading with less than 5 nmol/g of glucan, suggesting that the synergistic interactions between the cellulases and Xyl10As are very sensitive (Figure 6). The loading amounts of Xyl10Awt, Xyl10AdC, and Xyl10AdLC required to increase the glucan hydrolysis yield with Cel7A/Cel6A mixture by twofold were 0.67, 21.5, and 5.25-nmol/g of glucan, respectively. This suggests that Xyl10Awt was more effective in reducing the total enzyme loading for the degradation of WDM-RS glucan. The differences in the synergistic glucan hydrolysis among Xyl10Awt, Xyl10AdC, and Xyl10AdLC loadings seem to be related to the differences in the xylan hydrolysis yields (Figure 6). In fact, the glucan hydrolysis yield increased by the supplementation with the intact or mutant Xyl10As showed a good linear relationship with the xylan-hydrolysis yield (Figure 7).

The leverage ratio, as described by Kumar and Wyman, for Xyl10A supplementations in relation with the enhanced glucan hydrolysis of WDM-RS can be defined as the ratio of the percent increase in the glucose release to the percent increase in xylose release [6]. Interestingly, a two-stage leverage ratio was observed in the supplementation using Xyl10Awt for the range of 0 to 49.2 nmol/g of glucan after 24 h of hydrolysis, that is, 3.17 (*R*^2^ = 0.999) at less than 2% of xylan hydrolysis, and 1.62 (*R*^2^ = 0.999) at more than 2% of xylan hydrolysis (Figure 7a). The increase in the leverage ratio at lower supplementations of Xyl10Awt may correlate with the binding of Xyl10Awt to cellulose; consequently, Xyl10Awt would preferentially hydrolyze a minor xylan component that interrupts glucan hydrolysis. On the other hand, the leverage ratios for the supplementations of Xyl10AdC and Xyl10AdLC were calculated to be 1.86 (*R*^2^ = 0.993) and 1.66 (*R*^2^ = 0.991), respectively, and showed a linear relationship with the graph passing through the origin (Figure 7a). This result suggests that Xyl10AdC and Xyl10AdLC randomly target the xylan present in WDM-RS. The leverage ratios observed after 96 h of hydrolysis for the supplements of Xyl10Awt, Xyl10AdC, and Xyl10AdLC changed to 1.29 (*R*^2^ = 0.999, >4% of xylan hydrolysis), 2.01(*R*^2^ = 0.993), and 1.54 (*R*^2^ = 0.998, >2% of xylan hydrolysis), respectively (Figure 7b). Xyl10AdLC exhibited a slightly increased leverage ratio at less than 2% of xylan hydrolysis. The decreased leverage ratio observed for Xyl10Awt (>4% of xylan hydrolysis) may imply that the target for Xyl10Awt could have shifted to a larger xylan obstacle during the prolonged hydrolysis reaction.

## Discussion

It is generally recognized that xylanases have a role in the removal of xylan obstacles that prevent glucan hydrolysis by cellulases in a synergistic hydrolysis of pretreated lignocellulose. Earlier studies on modular xylanases have been focused on bacterial enzymes possessing xylan-binding CBM, such as family 2 [35,36], family 6 [37], and family 9 [38], and the importance of CBM2 for the synergistic hydrolysis of lignocellulosic substrates by bacterial GH11 xylanases has previously been demonstrated [35,36]. However, little attention has been paid to the effect of the cellulose-binding xylanase CBM1 for the hydrolysis of lignocellulose by a fungal cellulase system. This may be because all GH10 and GH11 xylanases in the cellulase system from *Trichoderma reesei*, which is a well-known industrial cellulase producer, are non-modular enzymes and show synergistic interactions with cellulases in the case of lignocellulosic substrates [13,39]. In the present study, using the characterizations of *T. cellulolyticus* Xyl10As with and without CBM1, we evaluated the role of xylanase CBM1 in the hydrolysis of glucan in the lignocelluloses. Further, our results revealed that the binding of Xyl10A to lignocellulose through CBM1 is important for the enhancement of hydrolysis of xylan and efficient synergistic hydrolysis of glucan in the lignocellulose.

In comparison to the Xyl10Awt, the hydrolytic activities of mutant Xyl10As without CBM1 were increased by 1.2- to 1.6-fold for soluble and insoluble xylans and chromogenic substrates, whereas the activities were decreased by 0.57- to 0.70-fold for WDM-RS (Table 1). On the other hand, xylan-binding CBM-deleted bacterial xylanases have been reported to show decreased hydrolytic activity not only for xylan in lignocellulose but also for the insoluble oat xylan [35,38,40]. These differences suggest that the cellulose binding by Xyl10A CBM1 is closely related to the efficient hydrolysis of lignocellulosic xylan. In addition, we confirmed that the Xyl10A CBM1 directly binds to cellulose by QCM-D (Figure 3). These observations support the hypothesis that the function of CBM is to increase the effective enzyme concentrations on the insoluble polysaccharide surfaces, to target the catalytic domain to the substrate [20,21]. On the other hand, a xylanase without CBM1 may be more suitable for the hydrolysis of the soluble hemicellulose fraction removed from the pretreated lignocellulose. Thus, it seems reasonable that various fungal cellulase systems naturally contain multiple GH10 and GH11 xylanases with and without CBM1 [18,19,41].

The hydrolytic activity of Xyl10AdC for WDM-RS xylan was relatively lower in comparison to the Xyl10AdLC without the Ser/Thr-rich linker region. The glycosylated linker of Xyl10AdC seems to have a negative effect on the hydrolysis of WDM-RS xylan. It has been suggested that Ser/Thr-rich linker regions in modular cellulolytic enzymes act as flexible connectors between subdomains, and the presence of linker glycosylation provides protection from proteases [42]. Recently, Payne *et al*. had reported that the glycosylated linkers enhance the binding affinity of the CBM1 alone by an order of magnitude [42]. The result of QCM-D analysis for Xyl10AdC exhibited that the Xyl10A linker without CBM1 shows weak interactions with cellulose (Figure 3). These observations predict that the Xyl10A linker weakly interacts with WDM-RS. However, the lack of CBM1 may lead to non-specific interactions with WDM-RS, resulting in a negative effect on the hydrolysis of WDM-RS xylan. Hence, the linker region without CBM1 is considered to be ineffective for hydrolysis of xylan substrates including lignocellulose.

There are a number of examples of synergistic interactions observed between cellulases and xylanase required for the hydrolysis of various pretreated lignocellulose substrates using crude or purified enzymes. It has been found that glucose yield increased almost linearly with the removal of residual xylose by the enzymes, despite the substantial differences in the relative yields for individual lignocellulosic substrates [6,12,17]. In this study, we revealed that the presence of Xyl10A CBM1 is not necessarily essential both for the synergistic interactions between cellulases and xylanases and for the linear relationship between increased hydrolysis of glucan and xylan for WDM-RS (Figure 5, Figure 7). However, it should be noted that an efficient synergistic hydrolysis was found in the interactions of the substrate with Xyl10Awt that has the highest xylan hydrolysis activity. Furthermore, the high leverage ratio at lower supplementations of Xyl10Awt seems to be closely associated with the specific adsorption of CBM1 of enzyme on the cellulose. Fungal CBM1s have a highly conserved structure and bind to similar targets present along the ridges of the crystalline cellulose [43]. Xyl10Awt is expected to bind in the proximity of Cel7A (or Cel6A) on the cellulose surface in WDM-RS, which enhances the susceptibility of the substrate to the action of cellulases due to preferential removal of xylan obstacles that interrupt the initial cellulase activity. In contrast, the CBM1-lacking Xyl10A mutants hydrolyze WDM-RS xylan non-specifically, resulting in lower efficiency of glucan hydrolysis and xylan removal. These results indicate that the binding of Xyl10A CBM1 to a cellulose surface has important implications not only for the enhanced hydrolysis of lignocellulosic xylan but also for the efficient synergistic activity with the cellulases.

On the other hand, it has been reported that non-productive adsorption of modular cellulolytic enzymes on lignocellulose substrates results in reduced hydrolysis efficiency [2]. Palonen *et al*. established a clear correlation between the presence of CBM1 in cellulases and the non-productive adsorption on the surface of lignin [44]. Qi *et al*. found that higher adsorption was observed for dilute acid treated wheat straw compared to dilute alkali treated sample [45]. In this study, we confirmed that most of the Xyl10Awt enzyme was adsorbed on a WDM-RS surface using a binding assay that measures the residual activity (data not shown). However, the high synergistic hydrolysis of WDM-RS at lower supplementations of Xyl10Awt suggests that Xyl10A CBM1 is specifically adsorbed on cellulose rather than on a lignin surface in the substrate (Figure 6). WDM-RS treated with mechanical fibrillation without any chemicals may reduce the non-productive adsorption of enzyme on a lignin surface.

Várnai *et al*. reported that CBMs in cellulases were not required for an efficient hydrolysis of lignocellulose with high consistency, although CBMs were more important in the catalytic performance of cellulases at low substrate concentration (1% w/v) [22]. The hydrolytic performance of cellulases without CBMs caught up with that of cellulases with CBMs at 20% (w/v) substrate concentration [22]. It should be noted that the role of Xyl10A CBM1 was evaluated at the relatively low substrate concentrations (3% w/w) in this study. Our results seem to support the importance of CBM1 at low substrate concentration. In addition, the hydrolysis of WDM-RS using the minimal cellulases and Xyl10A mixture was compared in the relatively low conversion of the glucan and xylan. The addition of accessory enzymes that hydrolyze the side groups in heteroxylan could improve the xylan hydrolysis yield in WDM-RS. Further work would be necessary to evaluate the effect of Xyl10A CBM1 for the higher hydrolysis yield of lignocellulose at high substrate concentration.

## Conclusions

We demonstrated that cellulose binding by Xyl10A CBM1 serves two roles in the synergistic hydrolysis of lignocellulose using Xyl10A and cellulases. First, it enhances the xylan hydrolysis activity by binding to cellulose, and second, it helps in the efficient removal of xylan obstacles that interrupt the cellulase activity due to a similar binding target of CBM1. Hence, the use of Xyl10A with CBM1 for the synergistic hydrolysis leads to the significant reduction in the enzyme loading used for the hydrolysis process. These findings may be applied to the synergistic hydrolysis of lignocellulose using other fungal hemicellulases and accessory enzymes possessing CBM1. The use of a combination of CBM1-containing modular cellulases, hemicellulases, and accessory enzymes present in the fungal cellulase system could contribute to further reduction of the enzyme loading for the hydrolysis of pretreated lignocellulosic substrates.

## Methods

### Strains, plasmids, and media

The *T. cellulolyticus* YP-4 uracil autotroph was selected for the production of recombinant proteins and was maintained on potato dextrose agar (Difco, Detroit, MI, USA) plates containing uracil and uridine at a final concentration of 1 g/L each [46]. The plasmid pANC202 [46], which contains the *pyrF* gene and the glucoamylase (*glaA*) gene promoter and terminator regions, was used in the construction of the plasmids pANC208 [19], pANC228, and pANC229 that were used for the expression of the Xyl10Awt, Xyl10AdC, and Xyl10AdLC proteins, respectively. *Escherichia coli* DH5α (Takara Bio, Kyoto, Japan) was used for the DNA procedures. The prototrophic transformants of *T. cellulolyticus* YP-4, that is, strains Y208 [19], Y228, and Y229, which express recombinant proteins Xyl10Awt, Xyl10AdC, and Xyl10AdLC, respectively, were maintained on MM agar (1% (w/v) glucose, 10 mM NH_4_Cl, 10 mM potassium phosphate (pH 6.5), 7 mM KCl, and 2 mM MgSO_4_, 1.5% (w/v) agar) plates [47]. The recombinant Xyl10As from these transformants were produced using a soluble starch medium containing 2% (w/v) soluble starch (Wako Pure Chemical Industries, Osaka, Japan) and 0.2% (w/v) urea as described in an earlier study [19].

### Plasmid construction and fungal transformation

The Xyl10Awt expression plasmid pANC208, which carries the 1.45 kb *xyl10Awt* genomic region from *T. cellulolyticus* CF-2612 chromosomal DNA (DDBJ accession number: AB796434) [19] was used as the template DNA to amplify the genes *xyl10AdC* and *xyl10AdLC*. A 1.35 kb DNA fragment containing the coding region of *xyl10AdC* was amplified by PCR using the forward primer 5′-ATT*GTTAAC*AAGATGACTCTAGTAAAGGCTATTC (*Hpa*I site in italics) and the reverse primer 5′-TAA*CCTGCAGG*CTAACCTGAGGTAGCGCTTGTGCTAGTC (*Sbf*I site in italics). A 1.23 kb DNA fragment containing the coding region of *xyl10AdLC* was amplified using the same forward and reverse primers, that is, 5′-AAT*CCTGCAGG*TTATAAGCCAGCAAGGATACCATAGTATG (*Sbf*I site in italics). The *xyl10AdC* expression plasmid pANC228 and the *xyl10AdLC* expression plasmid pANC229 were constructed by introducing each PCR fragment digested with *Hpa*I/*Sbf*I into the *Eco*RV/*Sbf*I site between the *glaA* promoter and terminator regions of pANC202. All ligated gene fragments and their ligation sites were verified by sequencing.

The plasmids pANC228 and pANC229 were transformed into protoplasts of *T. cellulolyticus* YP-4 by nonhomologous integration into the host chromosomal DNA [47]. Gene integration into the prototrophic transformants was verified using genomic PCR. Chromosomal DNA of the transformants was purified using the Gentra Puregene Yeast/Bact. Kit (Qiagen, Valencia, CA, USA). The strains showing high xylanase activity in the soluble starch medium were selected for the production of Xyl10AdC and Xyl10AdLC proteins and were designated as Y228 and Y229, respectively.

### Purification of recombinant Xyl10As

*T. cellulolyticus* strains Y208, Y228, and Y229 were grown in the soluble starch medium at 30°C, 200 rpm, for 96 h in an Erlenmeyer flask. The whole broth was centrifuged and the resulting supernatant was filtered through a 0.22-μm polyether sulfone membrane (Thermo Scientific, Rockford, Illinois, USA) under sterile conditions. The culture filtrate containing recombinant Xyl10As was stored at 4°C.

Enzyme purification was carried out using an ÄKTA purifier chromatography system (GE Healthcare, Buckinghamshire, UK) at room temperature. Purification of Xyl10Awt was performed by a Source 15Q (GE Healthcare) anion-exchange chromatography and a Source 15ISO (GE Healthcare) hydrophobic interaction column chromatography as described in an earlier study [19]. Xyl10AdC and Xyl10AdLC were purified by the same procedure as Xyl10Awt, except in this case where a Source 15Phe (GE Healthcare) hydrophobic interaction column was used instead of the Source 15ISO column. The protein applied to the Source 15Phe column was eluted with a linear gradient of 1.0 M to 0.0 M (NH_4_)_2_SO_4_ in 20 mM sodium acetate buffer (pH 5.5). The purity and size of the protein was analyzed by SDS-PAGE using NuPage 4-12% Bis-Tris gel (Invitrogen, Carlsbad, CA, USA). Glycosylation of proteins on an SDS-PAGE gel was detected by using the Pro-Q Emerald 300 glycoprotein stain (Invitrogen) following the manufacture’s instruction. All purified enzymes were preserved in a 20-mM sodium acetate buffer (pH 5.5) containing 0.01% NaN_3_ at 4°C. The protein concentration was determined with the Pierce BCA Protein Assay Kit (Thermo Scientific) using the bovine serum albumin as the standard (Thermo Scientific).

### Enzyme activity assays

Xylanase activity was measured by assaying the reducing sugars released after the enzyme reaction with 1% (w/v) xylan. The concentration of reducing sugars was determined using 3,5-dinitrosalicylic acid. The enzyme reaction was carried out in 50 mM sodium acetate buffer (pH 5.0) at 45°C for 10 min. Birchwood xylan (Sigma-Aldrich, St. Louis, Missouri, USA) and wheat arabinoxylan (Megazyme, Bray, Ireland) were used as the soluble xylan substrates. The insoluble xylan was prepared by boiling oat-spelt xylan (Tokyo Chemical Industry, Tokyo, Japan) for 30 min in distilled water and recovering the residue by centrifugation as described by Moraïs *et al*. [35]. The enzyme activity against WDM-RS was determined by assaying the reducing sugars released after 60 min of enzyme reaction with 3% (w/v) substrate at pH 5.0 (50 mM sodium acetate buffer) and 50°C. One unit of enzyme activity was defined as the amount of enzyme that catalyzed the formation of 1 μmol of reducing sugar per minute.

The enzyme activities against *p*-nitrophenol-based chromogenic glycosides (*p*-nitrophenyl-β-D-lactoside (PNP-lactose), *p*-nitrophenyl-β-D-cellobioside (PNP-cellobiose), and *p*-nitrophenyl-β-D-xyloside (PNP-xylose)) were determined at pH 5.0 in 50 mM sodium acetate buffer and at 45°C for 15 min using reaction mixtures including 1 mM PNP substrate. One unit of enzyme activity was defined as the amount of enzyme that catalyzed the formation of 1 μmol of *p*-nitrophenol per minute.

Kinetic parameters were determined using six concentrations of birchwood xylan (0.5 mg/ml to 16 mg/mL) at 45°C. Enzyme concentrations in the reaction mixtures (pH 5.0) were 24.1 nM (Xyl10Awt), 26.5 nM (Xyl10AdC), and 29.2 nM (Xyl10AdLC). The reaction was stopped at appropriate time intervals (2, 4, 6, 8, and 10 min), and the initial rates of xylan hydrolysis were determined accordingly. All experiments were repeated three times. The estimated kinetic parameters were obtained by fitting initial rates as a function of substrate concentration to the Michaelis-Menten equation using KaleidaGraph 4.1 (Synergy Software, Reading, PA, USA).

### Protein thermal shift assay

Thermal midpoint (*T*_m_) value of protein was determined by the fluorescence-based thermal shift assay using a real-time PCR detection system with the CFX Manager Program (CFX Connect, Bio-Rad, Hercules, CA, USA) as described in a previous study [46]. The protein sample containing the SYPRO orange dye (Invitrogen) was heated at 0.5°C per five seconds from 25°C to 95°C, and the fluorescence intensity (excitation/emission, 450 nm to 490 nm/560 nm to 580 nm) was measured every 0.5°C.

### Binding assay of Xyl10A for cellulose nanocrystal films

Cellulose nanocrystal film was prepared following the methodology described by Kumagai *et al*. [32]. CF11 cellulose powder (GE Healthcare) was fibrillated using a grinder (Crendipitor MKCA6-2, Masuko Sangyo Co., Ltd., Saitama, Japan) and subsequently using a high-pressure homogenizer (Masscomizer MMX-L100, Masuko Sangyo). The refined cellulose powder solution was ultrasonicated to improve its dispersibility and was spin-coated as cellulose nanocrystal film on the QCM-D gold sensors (QSX 301, Biolin Scientific, Stockholm, Sweden). The sensors were subsequently heated at 80°C for 10 min in an oven.

A QCM-D (Q-Sense E1, Biolin Scientific) was used for enzyme adsorption on the cellulose nanocrystal films. The sensor coated with the cellulose nanocrystal film was placed in 50 mM sodium acetate buffer solution (pH 5.0) overnight to allow it to swell completely. The swollen sensor was mounted in the QCM-D flow cell filled with the same buffer and was allowed to swell again until no appreciable frequency shifts were observed. The 250 μM enzyme solution in 50 mM sodium acetate buffer (pH 5.0) was introduced into the QCM-D flow cell with a peristaltic pump at a flow rate of 50 μL/min. The pump was stopped after 10 min when the buffer solution was completely replaced by the enzyme solution. The adsorption of enzymes onto the cellulose nanocrystal films was monitored for 2 h under static conditions. After that, only the buffer solution was introduced to rinse the system and follow the enzyme desorption. The temperature of the QCM-D flow cell was maintained at 40 ± 0.02°C during the measurements. The frequency changes at the fundamental resonance frequency (5 MHz) and its overtone frequencies (15, 25, 35, 55, and 75 MHz) were monitored simultaneously, and the third overtone (15 MHz) was used in the data evaluation. The frequency changes were converted into adsorbed mass density (μg/cm^2^) according to the Sauerbrey equation using the QTools software (Biolin Scientific) [48].

### Hydrolysis of pretreated rice straw

The WDM-treated rice straw was prepared using a Supermasscolloider MKZA10 (Masuko Sangyo), based on a previous report [34]. The rice straw slurry was introduced into the equipment, and the operation was repeated 20 times. The WDM-treated sample was freeze-dried and kept in a desiccator cabinet at room temperature until use. The composition (% dry weight) of structural carbohydrates in the pretreated rice straw was analyzed based on the standard NREL laboratory analytical procedure (LAP) [49] and was found to consist of 32.7% glucan, 19.4% xylan, 2.5% arabinan, and 1.5% galactan.

The hydrolysis of pretreated rice straw was carried out at 3% (w/v) solids loading in 50 mM sodium acetate buffer (pH 5.0). In the standard assay, the mixture consisting of Cel7A (38.9 nmol/g glucan) and Cel6A (44.5 nmol/g glucan) were replaced with various ratios of Xyl10Awt (0 to 98.4 nmol/g glucan), Xyl10AdC (0 to 108 nmol/g glucan), and Xyl10AdLC (0 to 119 nmol/g glucan) in a final volume of 1 mL. Bgl3A (6.17 nmol/g glucan) was added in all reaction mixtures. The total protein loading was 4.59 mg/g glucan. The reaction mixture was incubated at 45°C on a rotator. Cel7A, Cel6A, and Bgl3A were purified from *T. cellulolyticus* CF-2612 as described previously [24]. The hydrolysate of the pretreated rice straw was analyzed using a HPLC system equipped with a refractive index detector (RI-2031Plus, JASCO, Tokyo, Japan), an Aminex HPX-87P column (7.8 mm I.D. x 30 cm, Bio-Rad), and a Carbo-P micro-guard cartridge (Bio-Rad). The samples were eluted with water at a rate of 1 mL/min at 80°C. The glucan hydrolysis yield was defined as the ratio of the total equivalents of glucan hydrolyzed (glucose) to the total potential glucan available in the pretreated solids. The xylan hydrolysis yield was defined as the ratio of total equivalents of xylan hydrolyzed (xylose and xylobiose) to the total potential xylan available in the pretreated solids. All WDM-RS hydrolysis experiments were run in duplicates.

## Abbreviations

Bgl3A: glycosyl hydrolase family 3 β-glucosidase from *Talaromyces cellulolyticus*
Cel6A: cellobiohydrolase II from *Talaromyces cellulolyticus*
Cel7A: cellobiohydrolase I from *Talaromyces cellulolyticus*
CBM: carbohydrate-binding module
GH: glycosyl hydrolase
PNP-cellobiose: *p*-nitrophenyl-β-d-cellobioside
PNP-lactose: *p*-nitrophenyl-β-d-lactoside
PNP-xylose: *p*-nitrophenyl-β-d-xyloside
QCM-D: quartz crystal microbalance with dissipation monitoring
*T*_m_: thermal midpoint
WDM-RS: wet disc-mill treated rice straw
Xyl10A: glycosyl hydrolase family 10 xylanase from *Talaromyces cellulolyticus*
Xyl10Awt: recombinant intact xylanase
Xyl10AdC: CBM1-deleted xylanase
Xyl10AdLC: CBM1 and linker region-deleted xylanase
